# Effects of Ambidextrous Leadership on Employees’ Work Behavior: The Mediating Role of Psychological Empowerment

**DOI:** 10.3389/fpsyg.2022.862799

**Published:** 2022-05-16

**Authors:** Li Wang, Yuchen Sun, Jinzhi Li, Yunxia Xu, Meifen Chen, Xiaoyu Zhu, Dawei Wang

**Affiliations:** ^1^School of Psychology, Shandong Normal University, Jinan, China; ^2^School of Education, Wenzhou University, Wenzhou, China

**Keywords:** servant leadership, authoritarian leadership, organizational citizenship behavior, task performance, psychological empowerment

## Abstract

The complexity of today’s organizational environment increasingly requires leaders to think in a dynamic and flexible way to resolve contradictory issues. This study explored and compared the effects of servant leadership and authoritarian leadership on employees’ work behavior from the perspectives of ambidextrous leadership theory and social exchange theory, and further examined the mediating role of psychological empowerment. In this study, 315 employees from state-owned communication companies in Shandong and Zhejiang Provinces in China were selected as subjects, and path analysis was used to test the hypotheses. The results showed that servant leadership positively predicted organizational citizenship behavior and task performance. While authoritarian leadership negatively predicted organizational citizenship behavior and positively predicted task performance, psychological empowerment mediated the relationship between the two leadership styles and organizational citizenship behavior and task performance. Moreover, psychological empowerment and organizational citizenship behavior played a multiple mediating role between the two leadership styles and task performance. The theoretical implications of these findings for advancing the ambidextrous leadership theory in Chinese organizational contexts and practical approaches for corporate managers to effectively use ambidextrous leadership style were discussed.

## Introduction

In recent years, the world’s economic situation has been turbulent, and the domestic economic situation has become increasingly complex and volatile. More and more enterprises have begun to realize that employees’ work behavior is one of the important factors determining the success or failure of organizations. Employees’ work behavior that is often studied in current research includes organizational citizenship behavior (Ehrhart et al., 2015), task performance (Yin et al., 2018), and employee voice behavior (Hu et al., 2018), etc. It has been proven that leadership styles suitable for the company can effectively promote the positive work behavior of employees (Serban et al., 2015).

With the development of the world economy and culture, the enterprises’ pursuit of high performance and the employees’ need for humanistic care are in conflict, and leaders have to face many different or even mutually exclusive demands at the same time (Hou and Peng, 2019), such as employee-centered or task-centered, iron-fisted or gentle, etc. However, the previously explored single leadership style has become increasingly difficult to cope with the diverse needs of contemporary organizations. Thus, the concept of ambidextrous leadership has emerged. The ambidextrous leadership strategy is a dynamic process of tension resolution using contradictory and integrative thinking, which integrates two complementary leadership styles through a “both/and” mindset to form a new, more flexible, and adaptive leadership style. In the Chinese cultural context, the collision and friction between traditional leadership styles and modern employee needs is particularly prominent in organizational management situations. On the other hand, the traditional Confucianism and feudalism in China, which have been developed over thousands of years, promote the “the three cardinal guides and the five constant virtues” and “upper respect and lower inferiority” (Chen and Farh, 2010) from which the authoritarian leadership has emerged out of this thinking. It emphasizes dictatorship, demands absolute obedience from employees, and underlines high performance standards. Numerous researchers have argued that it best reflects the leadership style of Chinese society (Farh and Cheng, 2000; Pellegrini and Scandura, 2008; Zheng, 2018; Rui and Qi, 2021). Authoritarian leadership can play its role of efficiency, unity, and uniformity in a country like China where high-power distance and hierarchy prevails (Zhang et al., 2018). However, with the rise of modern humanistic management ideology and the new generation of employee power, employees are taking an increasingly important place in organizational management, and they are gradually not satisfied with the previous single material incentive. They begin to focus on spiritual needs for self-esteem, care, empowerment, self-actualization, and so on (Hou et al., 2014; Fang et al., 2020). Truly meeting the needs of employees has become an imperative issue for traditional Chinese leaders to consider in achieving effective leadership in the present day (Desky et al., 2020). In this context, servant leadership, which originated in the West and aims to serve employees and promote their potential development, is beginning to enter the Chinese perspective (Miao et al., 2014; Zhu and Wang, 2014; Vecchiotti, 2018; Long and Chen, 2020). As a result, a single leadership style may not be able to balance the traditional model with the diverse needs of modern development, and a diverse leadership mix adapted to the Chinese management context may resolve the conflicts and contradictions faced by organizations today.

Studies have shown that both authoritarian leadership and servant leadership are very important leadership styles in China, with the former being rooted in our cultural traditions (Qiu and Long, 2014) and the latter meeting the needs of China’s current economic and cultural development (Deng and Chen, 2012). Further, it has been demonstrated that servant leadership can promote employees’ beneficial work behaviors, such as organizational citizenship behavior (Newman et al., 2017), but there is still no consensus on whether authoritarian leadership can promote employees’ beneficial work behaviors. Some studies have shown that authoritarian leadership reduces employee voice behavior (Li and Yang, 2018) and organizational citizenship behavior (Zhang and Xie, 2017). However, others have shown that authoritarian leadership does not have a negative effect under high power distance conditions (Schaubroeck et al., 2016) and can even promote organizational citizenship behavior (Qiu and Yang, 2015). In the context of the great changes of the times, China is one of the few societies with a high-power distance. Hence, it is particularly important to explore the role of traditional authoritarian leadership and the emerging servant leadership in Chinese society, and which combination is more suitable for the development of Chinese companies in the framework of ambidextrous leadership theory.

In addition, we explored the mediating role of psychological empowerment between the two leadership types and employee work behavior. Psychological empowerment is a composite of people’s internal experiences when they feel empowered. It also includes four dimensions: meaning, self-efficacy, self-determination, and impact (Spreitzer, 1995). In this study, authoritarian leaders emphasize authoritarianism and personal centralization, which may reduce employees’ perceptions of self-determination and impact. In addition, harsh reprimands from leaders may also reduce employees’ self-efficacy, which may further negatively affect employees’ sense of meaningfulness at work and, subsequently, their perceptions of psychological empowerment. In contrast, servant leaders focus on employees’ growth and help them realize their potential as much as possible, which may increase employees’ self-efficacy and sense of meaning at work. Their appropriately empowering characteristics may also increase employees’ self-determination and impact, which in turn is beneficial to employees’ overall perception of psychological empowerment. We suggest that psychological empowerment may be a potential factor in explaining the differential impact of authoritarian vs. servant leadership on the same employee behaviors. Further, research has shown that psychological empowerment is an important psychological resource that employees acquire during social interactions and exchanges with leaders, and that it has a significant impact on employees’ work attitudes and behaviors (Li et al., 2006). Similarly, according to social exchange theory, different levels of psychological empowerment in interactions with authoritarian or servant leaders lead to different give-benefit analyses and different levels of rewarding behaviors. Therefore, this study suggests that the respective leadership characteristics of authoritative and servant leaders have important and different effects on employees’ perceptions of psychological empowerment, which in turn affects their work behaviors.

Therefore, based on social exchange theory, this study introduced psychological empowerment as a mediating variable to explore how servant leadership and authoritarian leadership affect employees’ work behavior, and further conducted a comparative study on their mechanisms of action.

## Literature Review and Hypotheses

### Servant Leadership, Authoritarian Leadership, and Employee Work Behavior

Originated from the West, Servant leadership prioritizes the welfare and growth of subordinates and pays attention to making employees work more effectively (Van and Nuijten, 2011), which has the characteristics of serving others and empowering (Jaramillo et al., 2015; Suyudi et al., 2020; Zhang et al., 2019). Based on the results of Liden et al. (2008), this study holds that servant leadership is a leadership style in which leaders discover the abilities, desires, and potential of employees and understand their characteristics and interests through communication and, based on that, help employees achieve their goals.

The effectiveness of authoritarian leadership has received extensive attention from researchers in the last two decades (Chen et al., 2018; Tu, 2018; Zheng et al., 2000). This leadership style is prevalent in collectivist and hierarchical cultures (Tian and Sanchez, 2017) and has both cultural origins and potential for development in China (Zhang et al., 2018). The main characteristics of authoritarian leadership is the emphasis on absolute authority and control of the leader and the absolute obedience of employees, as well as extremely high requirements for employees’ performance (Farh and Cheng, 2000; Jiang et al., 2017; Wang and Guan, 2018; Asim et al., 2021).

The collection of various types of behaviors exhibited by employees in the workplace is collectively referred to as employee work behavior (Zhang, 2015). In line with this, this study used organizational citizenship behavior and task performance as two indicators to measure and evaluated the level of employee work behavior. Organizational citizenship behavior is a series of informal cooperative behaviors that are not related to the formally incentive system and are not required within the role but are effective in improving organizational effectiveness as a whole. It is a kind of extra-role behavior (Organ et al., 2015). Task performance is an indicator that is directly related to work output and directly evaluates the results of employees’ work (Knight and Eisenkraft, 2015).

According to social exchange theory (Gouldner, 1960; Gergen, 1969), social life is a continuous series of resource transactions between two or more parties (Cropanzano et al., 2016), when subordinates receive support from leaders, they tend to reciprocate with positive work attitudes and performance, while when subordinates are intimidated by leaders, they tend to react negative reactions (Jiang et al., 2017). Servant leaders actively meet the growth needs of their employees, and employees will frequently demonstrate organizational citizenship behavior in return, which results in a mutually beneficial win-win process (Newman et al., 2017) and further improves employees’ performance (Chiniara and Bentein, 2016). On the contrary, in authoritarian leadership, although it can improve employees’ work efficiency, the control from the leader is likely to trigger employees’ negative emotions (Lin et al., 2010; Ma and Zhu, 2015), resulting in job burnout, which in turn reduces employees’ organizational citizenship behavior. In summary, we posited the following:

H1: Authoritarian leadership will negatively influence employee work behavior, reduce organizational citizenship behavior, and improve task performance.
H2: Servant leadership will positively influence employee work behavior, promote organizational citizenship behavior, and improve task performance.

Today’s complex and changing environment calls for leaders to adopt ambidextrous leadership strategies to balance the different demands within the organization (Hou and Peng, 2019). Ambidextrous leadership is a dynamic process of tension resolution using contradictory and integrative thinking (Luo et al., 2016), which requires leaders to look at employees’ demands and potential needs from two or more perspectives to respond appropriately. At present, Chinese enterprises are in the atmosphere of the integration of eastern and western cultures, and managers should not only draw wisdom from the long-standing Chinese traditional management style, but also learn the essence of the western scientific management system. However, authoritarian leadership and servant leadership are exactly the typical representatives of these two management philosophies. Servant leadership focuses on providing more services to employees, helping them grow and achieve their self-worth (Searle and Barbuto, 2011), while authoritarian leadership emphasizes task performance, control, and obedience (Cheng et al., 2004; Wang and Guan, 2018). If we integrate these two leadership styles based on the ambidextrous leadership theory, we can apply Chinese traditional leadership strategy of “employment of both kindness and severity” to the management of subordinates, achieving a good balance of humanistic care and strict requirements.

Therefore, servant leadership and authoritarian leadership can form an ambidextrous leadership combination, and four combinations are displayed according to the degree of difference between them (Table 1). Servant leadership and authoritarian leadership have different characteristics, and a proper interaction between them can have a unique and significant impact. High-service-high-authority leadership has the characteristics of the above two types of leadership style at the same time and is able to balance the various demands from the organization and the employees. It is not only able to focus on the employees, stimulate their potential, and promote their self-actualization, but also has a high demand for their performance, so it can simultaneously increase the organizational citizenship behavior and task performance of employees. Low-service-low-authority leadership, on the other hand, is exactly opposite of that, with no demands on employees and a lack of care and respect, which greatly increases the likelihood that employees are slack in work. High-service-low-authority leadership and low-service-high-authority leadership have their own emphasis. The former cares and focuses on exploring employees’ potential but lacks rigorous requirements for employees’ performance. Therefore, although it can positively predict employees’ organizational citizenship behavior and task performance, its positive predictive effect on task performance should be less than that of high-service-high-authority leadership, while the latter is focused on task performance and lacks humanistic care, which makes employees in a long-term high-pressure situation prone to burnout and, thus, show less organizational citizenship behavior. Hence, we proposed the following:

**TABLE 1:** Four combinations of ambidextrous leadership.

		Authoritarian leadership	
		High	Low
Servant Leadership	High	High-service-high-authority leadership	High-service-low-authority leadership
	Low	Low-service-high-authority leadership	Low-service-low-authority leadership

H3: High-service-high-authority leadership has a higher impact on organizational citizenship behavior and task performance than low-service-low-authority leadership. High-service-low-authority leadership has a higher impact on organizational citizenship behavior and task performance than low-service-high-authority leadership.

### The Mediating Role of Psychological Empowerment and Organizational Citizenship Behavior

Psychological empowerment is an intrinsic motivation of individuals, and it is a combination of personal perceptions of their work, including four dimensions: meaning, self-efficacy, self-determination, and impact (Thomas and Velthouse, 1990; Spreitzer, 1995; Wang and Zhang, 2011). Meaning refers to the work goals and values judged by individuals based on their own ideals and belief standards. The higher the match between the two, the stronger the sense of meaning perceived by individuals (Hackman and Oldham, 1980). Self-efficacy refers to people’s beliefs about their ability to perform their assigned work (Lawler, 1973; Bandura, 1986). Self-determination is people’s perception of the power of decision or control they have over their work, such as the ability to make their own decisions about work methods, work schedule, or work levels of effort (Deci et al., 1989). Impact is the perception that an individual has a significant impact on the strategy, management, and innovation of an organization at work (Abramson et al., 1978; Ashforth and Mael, 1989). Meta-analyses have shown that variables, such as leadership, are strongly associated with psychological empowerment (Seibert et al., 2011; Wang et al., 2020). When leaders implement more authorization behaviors in an organization, employees will perceive a higher level of psychological empowerment, and in turn, have a higher level of work engagement (Huertas-Valdivia et al., 2018), task performance (Spreitzer, 1995; Harris et al., 2009), organizational commitment (Liden et al., 2000), and organizational citizenship behavior (Chiang and Hsieh, 2012).

Servant leaders value care and respect for their employees, help them seize opportunities, and motivate them to achieve success by giving full play to their potential, which helps them increase their confidence and experience the sense of accomplishment and meaning brought by their work. In addition, empowerment characteristics displayed by servant leaders also provide a relaxing environment for employees to decide their own work behavior and participate in organizational management, which will lead to an increase in employees’ psychological empowerment (Liden et al., 2008; Neubert et al., 2008; Khan et al., 2021). According to social exchange theory, in the process of social interaction, individuals tend to analyze the price they pay and the reward they receive in a relationship. In addition, a satisfactory social relationship ensures a balance between the price and the reward. Therefore, in the face of a high level of perceived psychological empowerment from servant leaders, employees may exhibit positive work behavior in return (Allen et al., 2018). Numerous studies have shown that high levels of psychological empowerment are beneficial in motivating employees to perform more organizational citizenship behavior and improving their task performance (Chen et al., 2007; Harris et al., 2009; Wei et al., 2009; Wang et al., 2011; Newman et al., 2017).

Authoritarian leadership emphasizes a strict hierarchical relationship between leaders and employees. They tend to concentrate power in their own hands to show authority, closely monitor employees, and have little emotional communication with them, which can seriously weaken employees’ sense of control and decision making at work, thus reducing their perception of self-determination and self-influence. In addition, authoritarian leaders make high performance demands on their employees and directly reprimand and devalue those who do not perform well, which can also seriously undermine employees’ self-efficacy, make them lose confidence in completing their work, and not realize the meaning and value of their work. In other words, authoritarian leadership can weaken employees’ sense of psychological empowerment (Zhou and Liao, 2012; Yang et al., 2019). Also, based on social exchange theory, employees may reduce positive work behaviors in response to a low sense of psychological empowerment from authoritarian leaders. Employees with a low sense of psychological empowerment may not feel valued by the organization and reduce their attachment to and identification with the organization (Chiang and Hsieh, 2012), which in turn may reduce organizational citizenship behaviors. In addition, the lack of valuable resources, such as autonomy and decision-making power at work brought about by a low sense of psychological empowerment, may also increase employees’ uncertainty and job insecurity (Wang and Zhang, 2011; Wang et al., 2020), reducing employees’ work efficiency and negatively affects their task performance. Consistent with these arguments, we hypothesized the following:

H4a: Psychological empowerment will play a mediating role between servant leadership and organizational citizenship behavior, servant leadership and task performance.
H4b: Psychological empowerment will play a mediating role between authoritarian leadership and organizational citizenship behavior, authoritarian leadership, and task performance.

Leadership style has a significant impact on organizational citizenship behavior (Zhang and Xie, 2017). It has been shown that servant leadership has a positive influence on organizational citizenship behavior with its characteristics, such as valuing the needs and interests of employees (Walumbwa et al., 2010; Nobari et al., 2014; Alexandra et al., 2015), while authoritarian leadership has a negative effect on organizational citizenship behavior with its characteristics, such as authoritarian style and degradation of subordinates (Cheng et al., 2002; Liang et al., 2007; Zhang and Huai, 2012; Chan et al., 2013). However, organizational citizenship behavior can effectively overall improve organizational effectiveness (Borman and Motowidlo, 2014; Carpenter et al., 2014) and has a significant positive contribution to task performance (Podsakoff et al., 2009; Ren et al., 2013; Hermawan et al., 2020). Therefore, we expected the following:

H5a: Organizational citizenship behavior will play a mediating role between servant leadership and task performance.
H5b: Organizational citizenship behavior will play a mediating role between authoritarian leadership and task performance.

### The Serial Mediating Role of Psychological Empowerment and Organizational Citizenship Behavior

As mentioned above, both authoritarian leadership and servant leadership can influence the level of psychological empowerment of employees. While servant leadership enhances employees’ psychological empowerment, authoritarian leadership does the opposite. Employees who are exposed to servant leaders perceive a higher level of psychological empowerment, which further enhances their organizational commitment (Liden et al., 2000; Sunarsi et al., 2020), self-efficacy (Conger and Kanungo, 1988; Wang et al., 2020), and work efficiency (Li et al., 2006). According to social exchange theory, when employees perceive a higher level of psychological empowerment, their self-worth is affirmed and their need for respect is satisfied. In return, employees show more organizational citizenship behavior (Kim and Kim, 2013; Yang et al., 2015; Saira et al., 2020), which leads to an increase in overall organizational efficiency (Bateman and Organ, 1983). However, the efficient functioning of the organization will contribute to improving the efficiency of employees and ultimately to their task performance. In contrast, when faced with authoritarian leaders, employees have a lower sense of psychological empowerment, they perceive that they are under strict supervision and control, and their job autonomy and sense of work value are reduced. Similarly, based on social exchange theory, employees perceive that the support, respect, and recognition received from their leaders are not satisfied and, accordingly, they would reduce their organizational citizenship behavior (Settoon et al., 1996; Chan et al., 2013; Desky et al., 2020). This will have a negative impact on the overall organizational environment and climate (Organ et al., 2010), which in turn will be detrimental to the performance of employees, resulting in lower task performance. Consistent with these arguments, we expected the following:

H6: Psychological empowerment and organizational citizenship behavior will play a serial mediating role between servant leadership and task performance and between authoritarian leadership and task performance.

**FIGURE 1:**
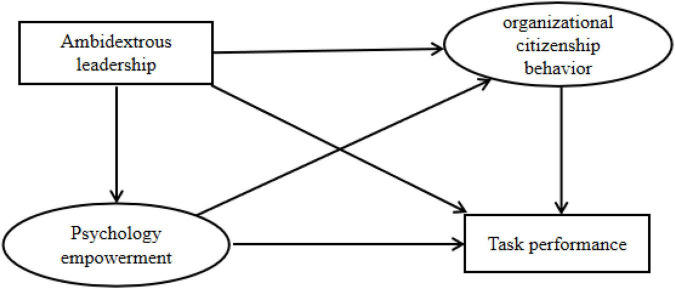
Hypothetical model.

In summary, based on social exchange theory, this manuscript constructed a multiple mediation model in which ambidextrous leadership influences task performance through psychological empowerment and organizational citizenship behavior, as shown in Figure 1.

## Methods

### Sample and Procedures

State-owned communication companies in Shandong and Zhejiang Province were selected to conduct the survey using a questionnaire method. The ethical approval for this study was obtained from the academic committee of Shandong Normal University and in accordance with the 1964 Declaration of Helsinki. In addition, the survey was approved by the company’s human resources department and the employees themselves, with signed informed consent form. Before measuring, the purpose of the survey was explained to the participants, emphasizing that the data were collected for scientific research only and that personal privacy was strictly protected to eliminate the participants’ concerns and ensure data quality.

A total of 400 employees participated in the survey and 358 questionnaires were returned, with a recovery rate of 89.5%. After eliminating unqualified questionnaires, data for 315 participants were valid, with a recovery rate of 79%. In terms of gender, 71.7% were females and 28.3% were males. Regarding age, 24.8% were 25 years old or younger, 46.9% were 26–30 years old, 15.6% were 31–35 years old, 7.3% were 36–40 years old, 5.4% were 41–50 years old, and 0% were above 51 years old. With regard to marital status, 29.2% were unmarried, 70.2% were married, and 0.6% were others. In relation to education level, 10.2% had a junior high school degree or less, 39.6% had a high school or junior college degree, 43.2% had a college degree, 7% had a bachelor’s degree, and 0% had a master’s degree or above. Regarding years of working, 0.6% of the participants had worked for less than 1 year, 22.5% for 1–3 years, 42.9% for 4–6 years, 22.9% for 7–9 years, and 11.1% for over 10 years.

### Measurements of Variables

#### Servant Leadership

The servant leadership scale was developed by Liden et al. (2008) and revised by Shen (2010). It consists of seven dimensions, namely, emotional comfort, helping employees grow and develop, putting subordinates first, creating value for the community, empowerment, ethical compliance, and conceptual skills, with sample items such as “My leader has a comprehensive understanding of our company and its goals.” It is involved in 8 items and are scored on a 7-point scale from 1 (completely disagree) to 7 (completely agree). The coefficient alpha for this scale in the study was 0.95.

#### Authoritarian Leadership

The authoritarian leadership scale is a sub-scale of the Paternalistic Leadership Scale developed by Fan and Zheng (2000), with sample items such as “My leader does not disclose information to us.” It includes 5 items and are scored on a 6-point scale from 1 (completely disagree) to 6 (completely agree). The coefficient alpha for this scale in the study was 0.81.

#### Organizational Citizenship Behavior

The organizational citizenship behavior scale was developed by Farh et al. (2004) and revised by Lv and Gu (2007), from which individual dimension with sample items, such as “You try to enrich yourself to improve the quality of your work,” and organizational dimension with sample items, such as “Take initiative to suggest improvements in your work,” were selected. It includes 15 items and are scored on a 5-point scale from 1 (completely disagree) to 5 (completely agree). The coefficient alpha for this scale in the study was 0.81.

#### Task Performance

The task performance scale developed by Tsui et al. (1997) and translated by Hou (2016) was used to evaluate employees’ productivity, quality, and quantity through 11 items, such as “I strive for excellence in my professionalism”. These 11 items are scored on a 7-point scale from 1 (completely disagree) to 7 (completely agree). The coefficient alpha for this scale in the study was 0.95.

#### Psychological Empowerment

The psychological empowerment scale was developed by Spreitzer (1995) and translated by Li et al. (2006). It is composed of four dimensions, namely, meaning, self-efficacy, self-determination, and impact, and its sample items included “I can decide for myself how to proceed to do my job.” It involves 12 items and are scored on a 5-point scale from 1 (completely disagree) to 5 (completely agree). The coefficient alpha for this scale in the study was 0.86.

#### Control Variables

Based on the selection of control variables in previous studies (Detert and Burris, 2007; Kamdar and Van Dyne, 2007), this manuscript used employees’ gender, age, education level, marital status, and working years as control variables.

## Results

### Common Method Bias

This study performed a common method bias test by Harmam single factor technique. The results showed that a total of 15 factors with characteristic roots greater than 1 were extracted, and the maximum factor variance interpretation rate was 30.74% (less than 40%). Therefore, there was no serious common method bias in this study (Podsakoff et al., 2003; Zhou and Long, 2004).

### Confirmatory Factor Analyses

This study used Mplus 8.0 for confirmatory factor analysis (CFA). As shown in Table 2, the measurement model fitted the data satisfactorily [*χ^2^/df* = 1.67, Root Mean Square Error of Approximation (RMSEA) = 0.05, Comparative Fit Index (CFI) = 0.90, Tucker-Lewis Index (TLI) = 0.90, Standardized Root Mean Square Residual (SRMR) = 0.06]. In addition, we further investigated several substitute measurement models and compared them with the five-factor model. The results indicate that the five-factor model fitted our data better than the other models, which suggests that our respondents are able to clearly discriminate the main constructs.

**TABLE 2:** Fit indices of each model for validation analysis (*N* = 315).

Models	*χ^2^/df*	RMSEA	CFI	TLI	SRMR
Five-factors: SL; AL; PE; OCB; TP	1.67	0.05	0.90	0.90	0.06
Four-factor: SL+AL; PE; OCB; TP	1.86	0.05	0.87	0.86	0.07
Three-factor: SL+AL+PE; OCB; TP	1.99	0.06	0.85	0.84	0.08
Two-factor: SL+AL+PE+OCB; TP	2.37	0.07	0.79	0.78	0.08
One-factor: SL+AL+PE+OCB+TP	2.97	0.08	0.70	0.68	0.10

*SL, servant leadership behavior; AL, authoritarian leadership behavior; PE, psychological empowerment; OCB, organizational citizenship behavior; TP, task performance; the symbol “+” indicates the combination of variables into one factor.*

### Difference Tests for Control Variables

The results of the difference tests for the control variables are shown in Table 3. Gender differed significantly on all variables; age and education differed significantly on the servant leadership, authoritarian leadership, psychological empowerment, and organizational citizenship behavior variables and not on the task performance variable; marital status differed significantly on the servant leadership and psychological empowerment variables, but not on the other variables; and years of experience were not differed significantly on all variables.

**TABLE 3:** Difference tests for the control variables.

Variables		Servant leadership	Authoritarian leadership	Psychological empowerment	OCB	Task performance
Gender	Male	5.59 ± 0.75	4.29 ± 1.08	4.07 ± 0.48	4.25 ± 0.44	5.78 ± 0.79
	Female	4.72 ± 0.79	3.64 ± 0.86	3.69 ± 0.51	3.92 ± 0.49	5.55 ± 0.81
	*t*	8.78***	5.55***	5.97***	5.63***	2.37**
Age	Under 25 years old	4.89 ± 0.82	3.73 ± 0.95	3.78 ± 0.52	3.93 ± 0.55	5.52 ± 0.94
	26–30 years old	4.75 ± 0.87	3.64 ± 0.97	3.73 ± 0.56	3.95 ± 0.49	5.64 ± 0.79
	31–35 years old	5.10 ± 0.76	4.03 ± 0.76	3.83 ± 0.46	4.11 ± 4.24	5.57 ± 0.69
	36–40 years old	5.83 ± 0.58	4.53 ± 1.12	3.97 ± 0.42	4.33 ± 0.35	5.75 ± 0.84
	41–50 years old	5.70 ± 0.62	4.34 ± 0.76	4.16 ± 0.50	4.30 ± 0.41	5.70 ± 0.75
	*F*	13.00***	3.26**	6.77***	5.75***	0.53
Marital status	Unmarried	4.79 ± 0.78	3.59 ± 0.85	3.71 ± 0.48	3.93 ± 0.51	5.57 ± 0.83
	Married	5.05 ± 0.90	3.92 ± 1.00	3.84 ± 0.55	4.04 ± 0.50	5.63 ± 0.80
	Others	4.44 ± 0.21	3.90 ± 0.42	3.42 ± 0.24	4.20 ± 0.57	6.00 ± 1.41
	*F*	3.13**	2.66	3.83**	1.68	0.38
Education level	Junior high school degree or less	5.71 ± 0.57	4.26 ± 0.84	4.05 ± 0.41	4.27 ± 0.40	5.80 ± 0.82
	High school/junior college degree	5.07 ± 0.80	3.93 ± 0.90	3.85 ± 0.50	4.04 ± 0.44	5.60 ± 0.77
	College degree	4.76 ± 0.90	3.69 ± 1.04	3.71 ± 0.56	3.93 ± 0.56	5.56 ± 0.86
	Bachelor’s degree	4.63 ± 0.83	3.46 ± 0.86	3.74 ± 0.57	4.00 ± 0.44	5.74 ± 0.72
	*F*	13.68***	4.31***	4.63***	4.26***	0.96
Years of working	Under 1 year	6.04 ± 0.10	4.60 ± 1.98	4.29 ± 0.41	4.47 ± 0.94	6.32 ± 0.19
	1–3 years	4.93 ± 0.84	3.72 ± 0.98	3.80 ± 0.54	3.92 ± 0.58	5.57 ± 0.85
	4–6 years	4.94 ± 0.83	3.79 ± 0.96	3.80 ± 0.53	4.02 ± 0.49	5.58 ± 0.83
	7–9 years	4.91 ± 0.85	3.85 ± 0.93	3.78 ± 0.51	4.07 ± 0.48	5.69 ± 0.69
	Over 10 years	5.22 ± 1.10	4.07 ± 1.03	3.81 ± 0.59	4.03 ± 0.40	5.63 ± 0.91
	*F*	1.62	0.45	1.13	1.21	0.65

Gender coded as (1 = male, 2 = female); age coded as (25 years old or younger = 1, 26–30 years old = 2, 31–35 years old = 3, 36–40 years old = 4, 41–50 years old = 5, above 51 years old = 6); education level coded as (junior high school degree = 1, high school or junior college degree = 2, college degree = 3, bachelor’s degree = 4, master degree or above = 5); marital status coded as (unmarried = 1, married = 2, others = 3); years of working coded as (worked for less than 1 year = 1, 1–3 years = 2, 4–6 years = 3, 7–9 years = 4, over 10 years = 5); **p < 0.01; ***p < 0.001; OCB, organizational citizenship behavior.

### Descriptive Statistics

As seen in Table 3, authoritarian leadership significantly and negatively correlated with psychological empowerment (*r* = −0.27, *p* < 0.01), organizational citizenship behavior (*r* = −0.34, *p* < 0.01), and task performance (*r* = −0.13, *p* < 0.01). Servant leadership had a positive and significant correlation with psychological empowerment (*r* = 0.57, *p* < 0.01), organizational citizenship behavior (*r* = 0.58, *p* < 0.01), and task performance (*r* = 0.48, *p* < 0.01). Furthermore, psychological empowerment significantly and negatively correlated with organizational citizenship behavior (*r* = 0.51, *p* < 0.01) and task performance (*r* = 0.52, *p* < 0.01). Organizational citizenship behavior had a positive and significant correlation with task performance (*r* = 0.61, *p* < 0.01). In addition, gender was significantly negatively related to servant leadership (*r* = −0.45, *p* < 0.01), psychological empowerment (*r* = −0.33, *p* < 0.01), organizational citizenship behavior (*r* = −0.32, *p* < 0.01), and task performance (*r* = −0.15, *p* < 0.01), and significantly positively related to authoritarian leadership (*r* = 0.31, *p* < 0.01). Age was significantly and positively correlated with servant leadership (*r* = 0.30, *p* < 0.01), psychological empowerment (*r* = 0.16, *p* < 0.01), and organizational citizenship behavior (*r* = 0.25, *p* < 0.01) and significantly and negatively correlated with authoritarian leadership (*r* = −0.24, *p* < 0.01), but did not correlate with task performance (*r* = 0.06, *p* > 0.05). Education level was significantly negatively related to servant leadership (*r* = −0.34, *p* < 0.01), psychological empowerment (*r* = −0.20, *p* < 0.01), organizational citizenship behavior (*r* = −0.18, *p* < 0.01), and significantly positively related to authoritarian leadership (*r* = 0.22, *p* < 0.01), but was not related to task performance (*r* = −0.06, *p* > 0.05). Marital status was significantly positively related to servant leadership (*r* = 0.10, *p* < 0.05) and organizational citizenship behavior (*r* = 0.10, *p* < 0.05) and negatively related to authoritarian leadership (*r* = −0.16, *p* < 0.01), but was not related to either psychological empowerment (*r* = 0.08, *p* > 0.05) or task performance (*r* = 0.03, *p* > 0.05). Years of working was not associated with servant leadership (*r* = 0.03, *p* > 0.05), authoritarian leadership (*r* = −0.09, *p* > 0.05), psychological empowerment (*r* = −0.02, *p* > 0.05), organizational citizenship behavior (*r* = 0.07, *p* > 0.05), and task performance (*r* = 0.02, *p* > 0.05) (Table 4).

**TABLE 4:** Means, standard deviations, and correlations (*N* = 315).

Variables	*M*	*SD*	1	2	3	4	5	6	7	8	9	10
1. Gender	1.72	0.45										
2. Age	2.21	1.07	−0.61**									
3. Education level	2.47	0.77	0.50**	−0.37**								
4. Marital status	1.71	0.47	−0.31**	0.45**	−0.14**							
5. Years of working	3.21	0.94	−0.24**	0.52**	−0.17**	0.39**						
6. Servant leadership	137.78	25.51	−0.45**	0.30**	−0.34**	0.10*	0.03	0.95				
7. Authoritarian leadership	15.92	4.83	0.31**	−0.24**	0.22**	−0.16**	−0.09	−0.27**	0.81			
8. Psychological empowerment	45.48	6.44	−0.33**	0.16**	−0.20**	0.08	−0.02	0.57**	−0.27**	0.90		
9. OCB	59.98	7.47	−0.32**	0.25**	−0.18**	0.10*	0.07	0.58**	−0.34**	0.51**	0.95	
10. Task performance	61.46	8.96	−0.15**	0.06	−0.06	0.03	0.02	0.48**	−0.13**	0.52**	0.61**	0.86

M, mean; SD, standard deviation. Gender coded as (1 = male, 2 = female); age coded as (25 years old or younger = 1, 26–30 years old = 2, 31–35 years old = 3, 36–40 years old = 4, 41–50 years old = 5, above 51 years old = 6); education level coded as (junior high school degree = 1, high school or junior college degree = 2, college degree = 3, bachelor’s degree = 4, master degree or above = 5); marital status coded as (unmarried = 1, married = 2, others = 3); years of working coded as (worked for less than 1 year = 1, 1–3 years = 2, 4–6 years = 3, 7–9 years = 4, over 10 years = 5); *p < 0.05; **p < 0.01; OCB, organizational citizenship behavior.

### Hypotheses Tests

First, we examined the effects of servant leadership and authoritarian leadership on employees’ work behavior (organizational citizenship behavior and task performance). The results showed that after controlling for demographic variables, servant leadership positively predicted organizational citizenship behavior (β = 0.55, *p* < 0.001) and task performance (β = 0.54, *p* < 0.001), supporting H1. In addition, authoritarian leadership negatively predicted organizational citizenship behavior (β = −0.26, *p* < 0.001) and positively predicted task performance with a borderline significance (β = 0.09, *p* = 0.05), supporting H2 to some extent.

**FIGURE 2:**
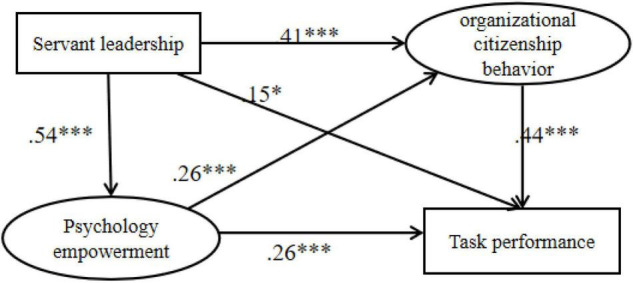
The serial mediation model between servant leadership and task performance. *p < 0.05, and ***p < 0.001.

Then, a sample of 1,000 times was taken using Bootstrap method to test the mediating role of psychological empowerment and organizational citizenship behavior. The results showed that servant leadership had a significant correlation with organizational citizenship behavior [β = 0.41, 95% CI (0.30, 0.53)], task performance [β = 0.15, 95% CI (0.03, 0.27)], and psychological empowerment [β = 0.54, 95% CI (0.45, 0.64)]. Furthermore, psychological empowerment had a significant correlation with organizational citizenship behavior [β = 0.26, 95% CI (0.15, 0.37)] and task performance [β = 0.26, 95% CI (0.16, 0.37)]. On the other hand, organizational citizenship behavior had a significant correlation with task performance [β = 0.44, 95% CI (0.33, 0.55)]. In light of these findings, H4a and H5a were supported (Figure 2).

**FIGURE 3:**
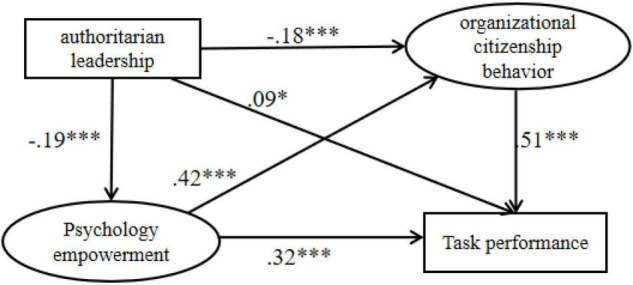
The serial mediation model between authoritarian leadership and task performance. *p < 0.05, and ***p < 0.001.

Meanwhile, authoritarian leadership had a significant impact on organizational citizenship behavior (β = −0.18, 95% CI [−0.28, −0.08]), task performance (β = 0.09, 95% CI [0.001, 0.18]) and psychological empowerment (β = −0.19, 95% CI [− 0.30, −0.08]). Furthermore, psychological empowerment had a significant impact on organizational citizenship behavior (β = 0.42, 95% CI [0.32, 0.52]) and task performance (β = 0.32, 95% CI [0.22, 0.42]). On the other hand, organizational citizenship behavior had a significant impact task performance (β = 0.51, 95% CI [0.41, 0.61]). In the light of these findings, H4a and H5a were supported (Figure 3).

### Interaction Analysis

**FIGURE 4:**
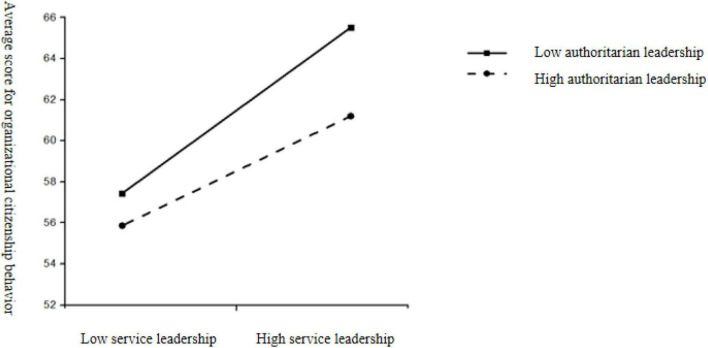
The interaction between authoritarian leadership and servant leadership on organizational citizenship behavior.

**FIGURE 5:**
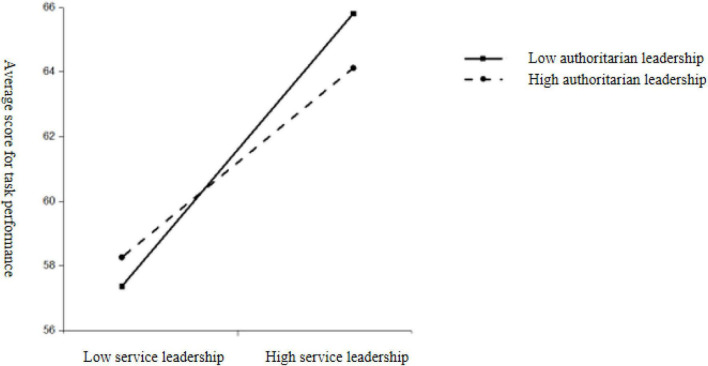
The interaction between authoritarian leadership and servant leadership on task performance.

The results of the one-way ANOVA analysis showed that the interaction of ambidextrous leadership styles was significant [organizational citizenship behavior: *F*_(3,315)_ = 38.35, *p* < 0.001, η^2^ = 0.193; task performance: *F*_(3,315)_ = 20.24, *p* < 0.001, η^2^ = 0.148]. When servant leadership was at the same level as authoritarian leadership, high-servant-high-authoritarian leadership scored significantly higher on both organizational citizenship behavior and task performance compared to low-servant-low-authoritarian leadership. When the levels of servant leadership and authoritarian leadership were not aligned, high-service-low-authoritarian leadership scored significantly higher than low-service-high-authoritarian leadership on organizational citizenship behaviors; while high-service-low-authoritarian leadership scored significantly higher than low-service-high-authoritarian leadership on task performance (as seen in Table 5, and Figures 4, 5).

**TABLE 5:** Organizational citizenship behavior and task performance scores under four ambidextrous leadership styles (*M ± SD*).

Ambidextrous leadership styles		*N*	Organizational citizenship behavior (*M ± SD*)	Task performance (*M ± SD*)
Servant Leadership	Authoritarian Leadership			
Low	Low	60	57.450 ± 7.720	57.367 ± 8.976
High		84	65.523 ± 5.649	65.810 ± 8.307
Low	High	98	55.867 ± 6.182	58.267 ± 8.164
High		73	61.206 ± 6.370	64.123 ± 7.597

## Discussion

### Theoretical Significance

Firstly, the results of this study indicated that servant leadership significantly and positively influences organizational citizenship behavior and task performance, and that authoritarian leadership negatively predicts organizational citizenship behavior and positively and marginally influences task performance. Leadership, as an important situational factor, has been the participant of much scholarly attention in terms of its impact on employee work behavior. Although servant leadership originates in the West, it is in line with the Confucian philosophy that “To a state, the people are the most important thing. The state comes second. The ruler is the least important,” and is closely related to the traditional Chinese culture. In addition, authoritarian leadership also has its roots in the system of patriarchal authority in the context of traditional Chinese society (Liu et al., 2018). Therefore, it is particularly relevant to explore the influence of these two leadership types on employee work behavior in the Chinese cultural background. This study shows that authoritarian and servant leadership have the same effect on employees’ task performance and different effects on employees’ organizational citizenship behavior, which can be explained by the social exchange theory. According to social exchange theory (Cropanzano et al., 2016), in the process of establishing economic and social relationships between organizations and employees, employees who receive rewards, such as care and support from servant leaders, will reciprocate by increasing their work commitment and fulfilling organizational role expectations, showing more organizational citizenship behavior and task performance (Chen et al., 2015). In contrast, authoritarian leadership emphasizes that authority cannot be challenged and requires absolute obedience from employees (Deng and Chen, 2012). Due to the authority of the leader, employees will ensure their own performance output and thus increase their own task performance. However, authoritarian leaders create an authoritative image, reduce social exchanges with employees, and induce employees’ negative emotions by exerting pressure, which can stimulate their workplace deviance, thereby reducing their organizational citizenship behavior. This finding is consistent with previous research (Neubert et al., 2016; Zhang and Xie, 2017) and validates that authoritarian and servant leadership have different effects on employee work behavior in the Chinese context. Meanwhile, it also provides a theoretical basis for the construction of these two types of leadership into an “ambidextrous” leadership.

Secondly, the results of this study also suggested that psychological empowerment plays a mediating role between servant leadership and organizational citizenship behavior and task performance, and also between authoritarian leadership and organizational citizenship behavior and task performance. That is, servant leadership increases employees’ perceptions of psychological empowerment, which in turn enhances their organizational citizenship behavior and task performance. According to social exchange theory (Jiang et al., 2017), employees will feel a higher level of self-efficacy and a sense of meaningfulness in their work when servant leaders support and care for them, which means that employees’ sense of psychological empowerment will increase, and they will show more organizational citizenship behavior and task performance in return. In contrast, authoritarian leaders emphasize the absolute authority of the leaders and the absolute obedience of the employees, reducing the employees’ sense of control and effectiveness at work, followed by a lower sense of psychological empowerment. In this situation, employees feel that they are not being rewarded as expected and will reduce their organizational citizenship behavior and task performance. This result not only reveals that psychological empowerment is an important transmission mechanism that can transfer positive or negative leadership behavior to employees’ workplace performance, but also provides a new perspective for future research, namely, to enhance the attention to the relationship between employees’ psychological empowerment and their own behavior.

Thirdly, this study found that the mediating role of organizational citizenship behavior is an inherent mechanism by which servant leadership influences task performance and authoritarian leadership. Previous research has demonstrated that organizational citizenship behavior has a contribution to task performance, and the results of this study reaffirm this view (Bruque et al., 2016). Besides, psychological empowerment and organizational citizenship behavior play a serial mediating role in the pathway of servant leadership affecting task performance, so as authoritarian leadership. Servant leadership increases employees’ psychological empowerment by causing them to perceive a higher level of autonomy and control at work. In return, employees exhibit more organizational citizenship behavior, which in turn further enhances task performance. However, the decrease of psychological empowerment brought about by authoritarian leadership makes employees show less organizational citizenship behavior and work less efficiently, reducing task performance and, to some extent, counteracts the positive direct effect of authoritarian leadership on task performance and diminishes its positive effect on task performance. This result further clarifies the mechanism of servant leadership on employees’ task performance and authoritarian leadership, and reveals the specific paths through which leadership behavior affects employees’ performance. Furthermore, previous studies have mostly examined employees’ organizational citizenship behavior as an outcome variable and have seldom explored their mediating role. However, the present study explores and verifies that organizational citizenship behavior can also serve as an effective mediator to transfer employees’ perceptions of psychological empowerment to their task performance, which fills a gap in existing research and provides ideas for future research.

Finally, based on ambidextrous leadership theory, this study combined servant leadership and authoritarian leadership to derive four types of ambidextrous leadership (Table 1), among which high-service-low-authority leadership has the best effect on employees’ work behavior. In addition, there is no significant difference with high-service-high-authority leadership. With the increasing level of China’s openness to the outside world, Chinese enterprises are accelerating their integration into the world development trend, learning and adapting to the rules of the international community. At the same time, the new generation of employees who make up the main market of China’s labor force are increasingly pursuing equality and disregarding authority. Hence, they already have a very different consciousness from the traditional society (Hou et al., 2014). Meanwhile the aging of China is becoming serious and the retirement age is being postponed again and again, which maintains people with traditional authoritarian ideas to still hold the voice in the organization. Therefore, it takes time to change the organizational culture. Against this background, the coexistence of two or even multiple leadership styles is more appropriate for today’s Chinese organizations than a single leadership style (Yu et al., 2014). The results of this study prove that servant leadership and authoritarian leadership can meet the requirements of ambidextrous leadership theory and achieve good complementarity. The high-service-high-authority leadership is able to integrate the human-centered caring culture with the organization’s demand for productivity improvement, achieving a win-win situation for both employees and the organization, which reveals the unique advantage of the traditional leadership strategy of “employment of both kindness and severity” in China’s traditional culture in the context of East-West cultural integration today. According to the contingency theory, the contingency combination and flexible application of servant leadership and authoritarian leadership is an important way to improve the effectiveness of leadership. Compared with the adherence to the authoritarian leadership or the total acceptance of the servant leadership from western culture, the integrated ambidextrous leadership is obviously more competitive.

### Practical Implications

Firstly, the findings found that two types of ambidextrous leaders, high-service-low-authority leaders, and high-service-high-authority leaders have unique advantages, suggesting that organizations should develop targeted leadership selection programs to screen leaders with these two characteristics. Secondly, emphasis should be placed on training existing leaders, as research has shown that leadership training can effectively improve leadership effectiveness (Zhang et al., 2015). Companies should pay attention to the development of leaders’ integrated thinking, so that they can flexibly use different combinations of leadership styles. Leaders should not only be employee-centered, give timely care to employees, and create conditions for their development, but also be aware of organizational efficiency, put forward appropriate performance requirements for employees, and urge them to improve efficiency and strictly comply with organizational norms. Thirdly, leaders should try to delegate and use employee assistance programs to improve employees’ perception of psychological empowerment and motivate them to engage in positive work behavior to improve performance. The serial mediating role of psychological empowerment and organizational citizenship behavior in the impact of ambidextrous leadership on task performance suggests that high levels of empowering behavior can improve task performance with half the effort. Therefore, leaders should attach importance to empowering behavior in their management, increase their attention to employees’ needs, recognize the equality between leaders and employees in work matters, accords employees with more powers of decision making and autonomy, and fosters an empowering and supportive organizational culture in the company. Finally, the manuals should be developed and disseminated in similar companies to expand the role of ambidextrous leadership and improve the overall effectiveness of the organization.

### Limitations and Suggestions for Research

First, all variables in this study were assessed on a self-assessment scale, which has the shortcoming of employees’ subjective factors influencing the assessment process. Future studies can adopt multiple time points and perspectives for data collection, such as collecting data in two phases or adding leadership evaluations or colleagues’ mutual evaluations. In addition, this study only preliminarily discusses the combination of servant leadership and authoritarian leadership, and the subsequent research can further discover the impact of the combination of other leadership styles. Moreover, this study did not consider the external environment factors when exploring the influence of leadership styles on employees’ work behavior. In fact, the organizational environment will inevitably influence this process. In future studies, we can introduce variables at both organizational and individual level and use techniques, such as cross-level structural equation modeling, to explore the interaction between individuals and the environment.

## Conclusion

(1) Servant leadership has a positive influence on organizational citizenship behavior and task performance, while authoritarian leadership has a negative influence on organizational citizenship behavior and a positive influence on task performance. Among them, psychological empowerment mediates the relationship between the two leadership types and organizational citizenship behavior and task performance, and psychological empowerment and organizational citizenship behavior play a serial mediating role between the two leadership types and task performance.
(2) Compared to the other two combinations of ambidextrous leadership types, high-service-low-authority leadership and high-service-high-authority leadership are shown to have unique advantages and a significant positive impact on employees’ organizational citizenship behavior and task performance.

## Data Availability Statement

The raw data supporting the conclusions of this article will be made available by the authors, without undue reservation.

## Ethics Statement

The studies involving human participants were reviewed and approved by the Academic Board of Shandong Normal University. The patients/participants provided their written informed consent to participate in this study.

## Author Contributions

LW: writing, revisions, and data analysis. YS: writing, data collection and analysis. JL: writing. YX: data analysis and revisions. MC: model building, data collection, and writing. XZ: data collection. DW: model building, writing, revisions, data collection and analysis, and supervision. All authors contributed to the article and approved the submitted version.

## Conflict of Interest

The authors declare that the research was conducted in the absence of any commercial or financial relationships that could be construed as a potential conflict of interest.

## Publisher’s Note

All claims expressed in this article are solely those of the authors and do not necessarily represent those of their affiliated organizations, or those of the publisher, the editors and the reviewers. Any product that may be evaluated in this article, or claim that may be made by its manufacturer, is not guaranteed or endorsed by the publisher.
